# Greek Tulips: Worldwide Electronic Trade over the Internet, Global Ex Situ Conservation and Current Sustainable Exploitation Challenges

**DOI:** 10.3390/plants10030580

**Published:** 2021-03-19

**Authors:** Nikos Krigas, Christos Lykas, Ioannis Ipsilantis, Theodora Matsi, Stina Weststrand, Mats Havström, Georgios Tsoktouridis

**Affiliations:** 1 Institute of Plant Breeding and Genetic Resources (IPBGR), Hellenic Agricultural Organization (HAO) Demeter, 57001 Thessaloniki, Greece; gtsok1@yahoo.co.uk or; 2 Department of Agriculture, Crop Production and Rural Environment, School of Agricultural Sciences, University of Thessaly, Volos, 38446 Magnesia, Greece; chlikas@uth.gr; 3 Soil Science Laboratory, School of Agriculture, Aristotle University of Thessaloniki, 54124 Thessaloniki, Greece; iipsi@agro.auth.gr (I.I.); thmatsi@agro.auth.gr (T.M.); 4 Gothenburg Botanical Garden, Carl Skottsbergs Gata 22A, SE-413 19 Gothenburg, Sweden; stina.weststrand@vgregion.se (S.W.); mats.havstrom@vgregion.se (M.H.); 5 Theofrastos Fertilizers, Irinis & Filias, Examilia Korithias, 20100 Korinthos, Greece

**Keywords:** biodiversity, botanic gardens, e-commerce, Greek flora, Liliaceae, phytogenetic resources, seed banks, *Tulipa*

## Abstract

From an ornamental viewpoint, tulips are famous clonally propagated crops. This research focuses on 15 wild-growing Greek tulip species including 11 range-restricted species, i.e., six Greek endemics and five Balkan or Aegean endemics and subendemics, among which seven are currently threatened with extinction (two Critically Endangered, three Endangered and two Vulnerable). An overview of the global electronic trade over the internet is presented herein for these valuable phytogenetic resources in an attempt to define the extent of their commercialization (25 nurseries in three countries, mainly bulb trade at various prices) with concomitant conservation implications. In the frame of the repatriation initiatives launched, their global ex situ conservation is overviewed according to the PlantSearch facility of the Botanic Gardens Conservation International (materials from 15 species stored in 41 botanic gardens of 14 countries). The results of this study on the Greek tulips showed that there are both well-established value chains and gaps in the market regarding the “botanical tulips”; revealed the compromised effectiveness of ex situ conservation for the majority of them; raised conservation concerns related to authorized access to these wild phytogenetic resources; and indicated that their future utilization should comply with the provision of national and international legislation. All these are envisaged and discussed within the framework of the newly launched research project TULIPS.GR which aims to be the pilot establishment of a national collection regarding all Greek tulips (currently holding 38 accessions of 13 species, including almost all of the threatened ones). The project’s scope is to enable the creation of a sustainable value chain for the Greek tulips with authorized collections, sustainable conservation schemes, production of DNA barcoded propagation material, species-specific propagation and cultivation protocols, mycorrhizal investigations, field studies, applying innovative precise soil/foliar fertigation, and investigation of the postharvest treatment of fresh cut flowers, promoting networking and synergies with producers and associations in Greece and abroad.

## 1. Introduction

The electronic plant trade over the Internet has been largely facilitated by social media and communication platforms resulting to date in a popular new way of easy plant trade worldwide [1,2,3,4,5,6,7,8,9,10,11]. This kind of uncontrolled e-trade can have devastating effects to wild-growing populations, and thus may undermine conservation efforts both nationally and globally due to unrestrained overexploitation of local single-country endemics and/or threatened species [2,3,9]. Theoretically, when the international e-commerce of plants is performed under the provisions of the Nagoya protocol and the EU Directive 511/2014 which regulate sovereign rights over phytogenetic resources and concomitant Access and Benefit Sharing mechanisms for their sustainable exploitation, it may secure local phytogenetic resources, also offering support to domestic subsistence economies [6,8,9,10,11,12].

Due to severe anthropogenic disturbance of wild habitats and under the threat of climate change, ex situ plant conservation serving as back-up of in situ conservation has become increasingly important over the last decades for the conservation of phytogenetic resources [13,14,15,16,17]. In this framework, botanic gardens (BGs) and seed banks (SBs) play a pivotal role in achieving effective species’ conservation [13,14], with their greatest concentration occurring in northern temperate regions (especially Europe) [13,14]. However, the need for such conservation initiatives is more intense in floristically diverse southern countries within this geographical context, and especially in the Mediterranean region which hosts many threatened plants [15,18,19,20,21], and in countries which are relatively poorly resourced in conservation facilities compared to less diverse northern ones [14]. Furthermore, BGs and SBs often house in man-made environments several socioeconomically important plants from around the globe. Therefore, they may be considered as sources of valuable donor material and species-specific propagation and cultivation know-how, activating considerable opportunities for the sustainable exploitation of phytogenetic resources in the short-term, medium-term or long-term [12,22,23]. This applies to major crop plants, including widely appreciated ornamentals such as tulips, but also to neglected and underutilized plants such as local endemics confined to specific regions [12,22].

In general, the domestication of wild-growing plants with interesting properties associated with plant rarity or endemism (uniqueness) has been extremely appreciated by the ornamental-horticultural sector, since the latter is always in quest of and sourcing for unique new crops with attractive features [6,22,24]. In this context, tulips are famous ornamental plants worldwide since the Middle Ages [11,24], and currently in Europe they are associated with a turnover of about half a billion euros yearly with increasing trends [24]. As a result of this long-lasting appreciation, to date there are hundreds of traded hybrid tulip varieties raised through breeding strategies from a few ancestral East Mediterranean and/or Asiatic species to satisfy commercial needs, as well as several nonimproved species of the genus *Tulipa* that are used for ornamental reasons, mainly by plant enthusiasts and garden lovers.

In line with the foregoing studies related to the electronic trade [3,7,11], the ex situ conservation of focal endemic plants [14] and the sustainable exploitation of neglected and underutilized phytogenetic resources [12], the study herein focuses on the wild-growing tulips of Greece. These phytogenetic resources comprise 15 *Tulipa* spp. which are nationally protected by the Greek Presidential Decree 67/1981, among which six are confined to the Greek territory (single-country endemics) and five are local Balkan or Aegean endemics and subendemics extending to adjacent countries. Seven of them are assessed as threatened (two Critically Endangered, three Endangered and two Vulnerable) according to the criteria of IUCN (International Union for the Conservation of Nature) [25,26,27]. In an attempt to explore the extant value chain associated with the Greek tulips internationally and to define the extent of the current commercialization of Greek tulips over the internet, an overview of their global electronic trade is presented herein and concomitant conservation implications are discussed. To promote the repatriation initiatives of well-documented plant material acquired in Greece but housed abroad, the global ex situ conservation of Greek tulips is overviewed according to the PlantSearch facility of the Botanic Gardens Conservation International (BGCI). These efforts are envisaged in the frame of the newly launched efforts to create a national collection of wild Greek tulips, attempting to pave the way for the creation of a Greek national tulip collection and a sustainable value chain associated with them.

## 2. Results and Discussion

### 2.1. Electronic Trade of Greek Native Tulips

The traded Greek tulips are often classified by the nurseries involved as “botanical tulips” to contrast with commercialized hybrid tulips, thus reflecting their distinct origin. Botanical tulips (e.g., https://www.gardenia.net/plant/tulipa-saxatilis-lilac-wonder-botanical-tulip, etc. (accessed on 13 March 2021)) are ancestors of cultivated tulip hybrids and they are almost nonimproved by breeding strategies. Being evolved to adapt to natural conditions, quite often these botanical tulips are hardier and easier to grow compared to hybrids. When these are attractive but also rare, protected, and local endemic of specific regions, they are perceived by people as uncommon to them (or “exotic” due to their distant origin from other regions than those of the consumers), thus presenting a “new” attractive choice for the ornamental industry which is always “thirsty” for novel beauties [12].

Surprisingly, though not unexpectedly if previous research is considered [3,7,11], the UK dominates the electronic market over the internet related to the tulip bulb trade of Greek species, and this trend is followed by Dutch nurseries. Perhaps this dominance reflects the long-standing tradition of home gardening in UK and the developed industry [7]. In total, 11 Greek tulip species are readily available as bulbs over the internet; 10 of them are traded globally by 13 UK nurseries, five species are supplied from nine Dutch nurseries and two from others that are located in the USA (Table 1).

Bulbs of most of the Greek tulip species are actually sold out every season according to the nurseries’ websites (namely stated as “out of stock”). As anticipated [3,7,11], it was not possible to determine the extent of this e-trade in terms of quantities dispatched [3,11,17]. Regularly, on the webpages of more than half of the nurseries involved there are specific “want lists” or “wish lists” (>15 nurseries) to inform customers promptly in order for the nurseries to be able to schedule and dispatch the requested materials. On the other hand, this also means that the customer may request a certain species that might not be available on the nurseries’ websites. Based on these demands, the nurseries are supposed to try to find and deliver the requested materials, either from collaborating nurseries or elsewhere [3,7,11]. None of these nurseries inform, however, about the original provenance of the traded materials. In this way, it is not known whether the traded plants are raised yearly from initial cultivated stocks, or if these materials have been sourced or collected directly or partly from wild habitats (some of them or sometimes). Of course, no nursery webpage states how and when the initial plant material (mother plants) was obtained. At least some of the nurseries’ websites state that they make regular collecting trips around the world [11], e.g., https://seedsofpeace.info/ (accessed on 13 March 2021). To this end, it has been suggested that perhaps a network of local collaborating collectors is activated upon such requests to try to find the selected materials, however without any authorization to collect and purchase tulip bulbs and/or seeds (see discussion in [3,7,11]). When true, such pressure exerted on local endemic and/or threatened species may have devastating effects on wild-growing populations.

There is a wide variety of prices for dispatched quantities (5–1000 bulbs) ranging from £0.16–0.50/bulb for the protected *T. bakeri* A. D. Hall which is a Cretan single-island endemic (e.g., https://www.rosecottageplants.co.uk/tulipa-bakeri-lilac-wonder/p525 (accessed on 13 March 2021)) to £1.50/bulb and £2.60/bulb for *T. clusiana* DC. and *T. australis* Link, respectively (1 £ = 1.16 € as of 1/3/2021).

Greek tulip seeds are rather hard to find over the internet, and when these are located, they are quite a lot more expensive in respect to bulbs. For example, the Mediterranean *T. australis* is currently available as seeds (packet) in UK at £2.85. The Irano-Turanian *T. agenensis* DC. as well as the protected and threatened (Table 1) Cretan endemics *T. bakeri* https://seedsofpeace.info/product/tulipa-bakeri/ (accessed on 13 March 2021)) and *T. cretica* Boiss. & Heldr. (https://seedsofpeace.info/product/tulipa-cretica/ (accessed on 13 March 2021)) are to be found only in an Israeli nursery; seeds of each of these three tulips are sold at 5 € for 10, 12 or 15 individual seeds, respectively. This high price of 0.3–0.5 € per individual seed is almost equivalent sometimes to that of the traded individual bulb (compare with above-mentioned prices of *T. bakeri*).

To date, we detected no availability over the internet regarding seeds for 11 of the Greek tulips (73.33%). Likewise, no bulbs were found as currently traded for 26.67% of the Greek tulips, i.e., the protected local Balkan subendemic *T. bithynica* Baker, the protected and threatened Greek endemic *T. goulimyi* Sealy & Turrill and the protected local Balkan endemics *T. rhodopea* (Velen.) Velen and *T. scardica* Bornm. (Table 1).

### 2.2. Ex situ Conservation of Greek Native Tulips

Overall, our survey showed that all 15 Greek tulips can be found in the ex situ facilities of 41 botanic gardens (BGs) located in 10 European countries (10 BGs in UK), but also in the USA (12 BGs), Canada, Australia and New Zealand (Table 1). Species-wise, *T. saxatilis* Spreng., *T. clusiana* and *T. orphanidea* Heldr. are most widespread in the BG community, appearing in 26, 22 and 15 institutions of 12, 9 and 8 countries, respectively, followed by *T. agenensis* DC., *T. cretica* and *T. hageri* (each in 9 institutions of 5–6 countries).

Nevertheless, not all of these collections originate from Greek phytogenetic resources, and it is largely unclear whether these stored genetic materials originate directly from wild-growing populations or if they were obtained through exchanges with other BGs, thus representing mere duplications of clonally propagated material. Such information can only be sought through specific requests to individual BGs. The most prominent representation of Greek tulips (Table 1) can be found in the Gothenburg BG (14 species), the Cambridge University BG (7) and the Royal BGs of Edinburgh (7). Although the protected and threatened *T. undulatifolia* Boiss. is to be found in eight BGs of seven countries, the ex situ conservation of the rest of the Greek tulips of conservation concern (threatened and/or range-restricted species) is deemed as rather ineffective [14]. This holds true for the threatened local endemics *T. doefleri* Gand. and *T. goulimyi* as well as the local Balkan (sub-) endemics *T. bithynica*, *T. rhodopea*, and *T. scardica* which are found only in two BGs worldwide (Table 1).

### 2.3. Current Sustainable Exploitation Challenges

**Figure 1:**
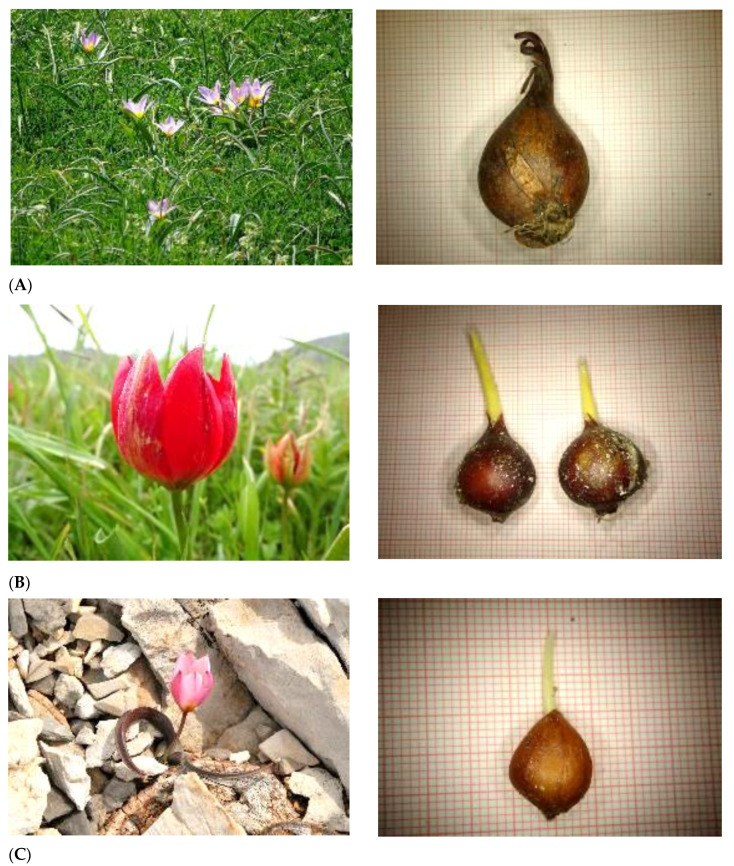
Wild-growing Greek tulips endemic to the island of Crete and individual bulbs collected for ex situ conservation in the frame of TULIPS.GR: (**A**) *Tulipa bakeri* and (**B**) *T. doerfleri* assessed as Critically Endangered [27], and (**C**) *T. cretica* (photo: V. Papiomytoglou) assessed as Endangered [27].

**Figure 2:**
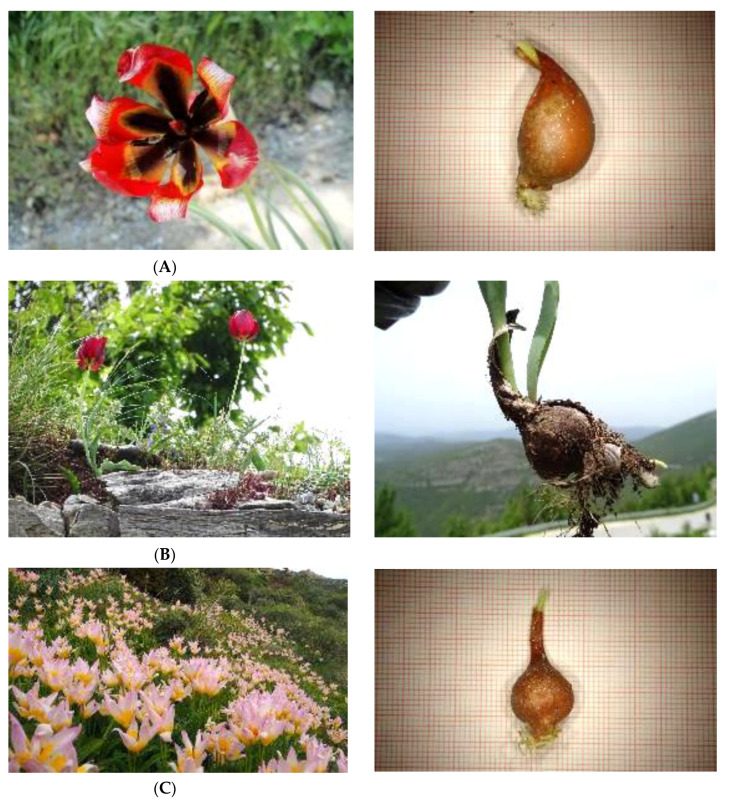
Wild-growing, range-restricted Greek tulips and individual bulbs collected for ex situ conservation in the frame of TULIPS.GR: (**A**) *Tulipa bithynica* and (**B**) *T. rhodopea* endemic to southern Balkans and/or Anatolia (Balkan subendemic); (**C**) *T. saxatilis* (photo: M. Avramakis, Natural History Museum of Crete) endemic to major south Aegean islands (Crete, Karpathos, Rhodes) and south-western Turkey (south Aegean subendemic).

**Figure 3:**
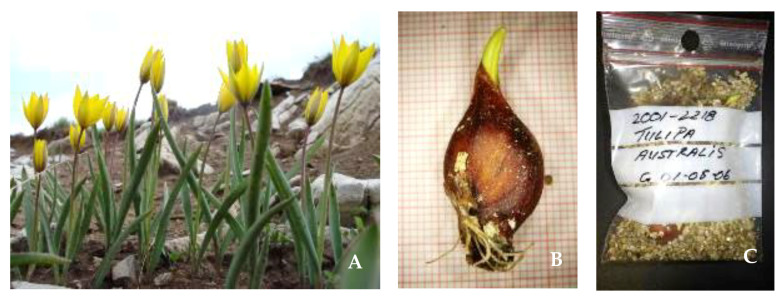
(**A**) Wild-growing individuals of the Mediterranean *Tulipa australis* at the summit area of Mt Vermion, Northern Greece; (**B**) underground individual bulb; and (**C**) repatriated well-documented bulbs of the same species from the Gothenburg Botanical Garden, Sweden that were originally collected close to the summit area of Mt Vourinos, northern Greece in 2001.

**Figure 4:**
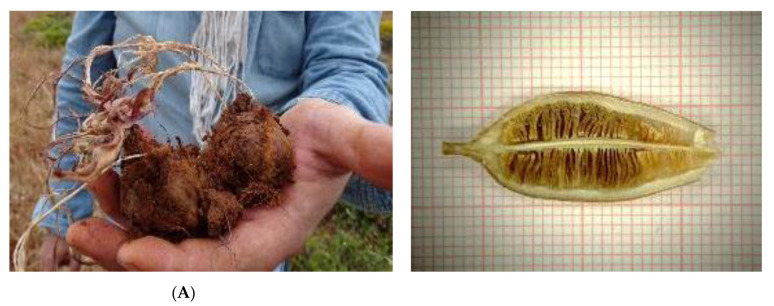

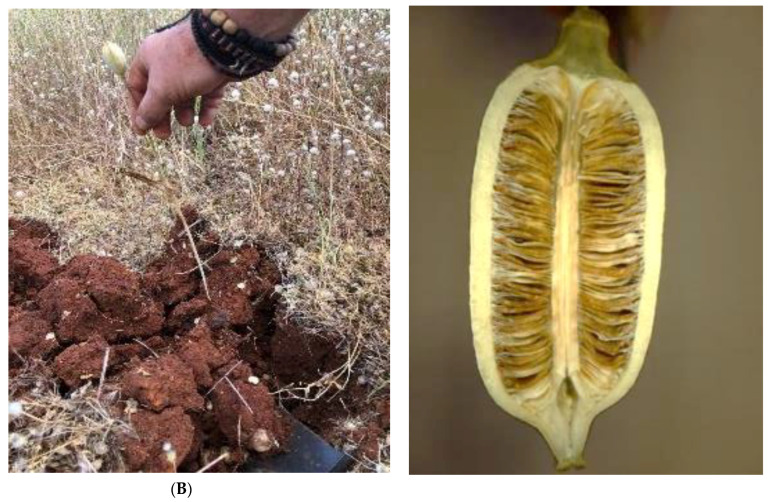
Dormant bulbs and capsules with ripe seeds collected for ex situ conservation from wild-growing and protected populations of Greek tulip species assessed as threatened with extinction [25,27]: (**A**) *T. goulimyi*, a local endemic of Peloponnese, Kythira-Antikythira and west Crete, assessed as Endangered [27]; (**B**) *T. undulatifolia*, an endemic to Southern Balkans and west and central Anatolia (Turkey), assessed as Vulnerable [25].

The newly launched research project TULIPS.GR is focused on the ex situ conservation, domestication and sustainable exploitation of the 15 wild-growing Greek tulips. To this end, an integrated approach is followed beginning with well-documented collections performed with an authorized collection permit issued by the Greek ministry of Environment and Energy. Initially, as a first step towards the creation of a national collection of Greek tulips, the project has already documented and incorporated under ex situ conservation 12 accessions of eight wild-growing Greek tulips which were collected directly from wild-growing populations (Table 1, Figure 1, Figure 2, Figure 3 and Figure 4), prioritizing the nationally threatened species [25,26,27]. In an attempt to increase the genetic variability of Greek tulip germplasm, the project aims to collect over the next three years selected propagation material from as many spontaneous populations as possible, covering all wild-growing Greek tulip species.

Additionally, a repatriation initiative has been launched through the Balkan Botanic Garden of Kroussia (BBGK), Institute of Plant Breeding and Genetic Resources (Agricultural Organization Demeter) concerning the range-restricted and local endemic species of Greek tulips. This procedure involved personalised letters sent by e-mail to the curators of a dozen BGs holding Greek tulip species. However, almost none responded positively in this call by informing explicitly about the stored materials. However, the Gothenburg BG in Sweden responded immediately to this call by sending 20 well-documented accessions of nine Greek tulip species for repatriation in the BBGK; these materials were added to the new Greek national collection (Table 1, Figure 3). The repatriated materials from Sweden had been originally collected in the Greek territory at earlier times (1984–2009) by various famous botanists (e.g., A. Strid), and are currently under duplicated ex situ conservation for safety reasons (Swedish and Greek BGs). Some of the BGs contacted to date are not yet in a position of repatriating bulbs or seeds from tulips of Greek origin; however, some of these (e.g., Cambridge University BG) have already flagged their materials in order to be able to respond in the future. Additional requests will be made in the near future to more BGs holding accessions of tulips collected from Greece.

Furthermore, in an attempt to avoid trivial trial-and-error losses during propagation and cultivation procedures with domestic valuable phytogenetic resources, the selected material of five Greek tulip species (*T. australis, T. bakeri, T. cretica, T. orphanidea, T. saxatilis*) has already been purchased from specialized nurseries. This material has been obtained for e-commerce verification serving documentation purposes, and it will be used for basic experimentation and species-specific comparisons with wild type materials by molecular barcoding in the frame of TULIPS.GR (Table 1).

Overall, 38 documented accessions of 13 Greek tulip species are currently under evaluation in the frame of TULIPS.GR, 82% originating from the wild-growing populations of Greece. The experimentation in progress includes the development of species-specific propagation and cultivation protocols, study of mycorrhizal diversity, foliar symptoms diagnostics, fertilization regimes with innovative domestic organic fertilizers produced by Theofrastos company (https://theofrastos.com/en/theofrastos-liquid-organic-fertilizers/; accessed on 13 March 2021), as well as the development of a strategy for postharvest treatment.

## 3. Materials and Methods

Chorology, endemism, and protection status of the 15 wild-growing Greek tulips were retrieved from the official Vascular Flora of Greece: an annotated checklist (http://portal.cybertaxonomy.org/flora-greece/; accessed on 13 March 2021). For species’ extinction risk assessments using the IUCN criteria (at least the IUCN criteria A and B), we consulted different sources at global (www.iucnredlist.org; accessed on 13 March 2021) and national scale [25,26,27].

We used previously established methodologies to detect the extent of the electronic commerce associated with Greek tulips [3,7,11]. In brief, we conducted individual searches using the scientific names of the Greek tulip species and/or their main synonyms according to the official website of the Vascular Flora of Greece: an annotated checklist (http://portal.cybertaxonomy.org/flora-greece/ (accessed on 13 March 2021)). During each quest in the Google platform (www.google.com (accessed on 13 March 2021)), we recorded different vendors (e-shops of specialized nurseries), prices and type of materials (bulbs, seeds) traded over the internet, as appearing in the first 10 pages of the search engine [11]. Additionally, we used in the same way the Plant Finder application (https://www.rhs.org.uk/plants/search-form (accessed on 13 March 2021)) of the Royal Horticultural Society webpage, as well as the Plant Information Online webpage (https://plantinfo.umn.edu/ (accessed on 13 March 2021)) [3,7,11]. Then, we aggregated all data retrieved till 13 of February 2021.

To assess the current situation (as of 20 of February 2021) of the ex situ conservation of Greek tulips in botanic gardens (BGs) worldwide, the PlantSearch facility of the Botanic Gardens Conservation International (BGCI; http://www.bgci.org (accessed on 13 March 2021)) was used as previously described [14,28]. In brief, we recorded the names and the number of BGs offering ex situ conservation to Greek tulip species as well as the countries where these are located. It should be mentioned that the BGCI’s PlantSearch facility does not provide data on the number of accessions belonging to a specific taxon in various BGs, but only the number of BGs where a taxon is to be found. Accepting that accessions of the different BGs differ between each other, this number is equivalent to the minimum number of extant accessions of a taxon in the BGs worldwide [14,28].

Access to wild-growing tulip populations and plant collections in the Greek territory (Figure 1, Figure 2, Figure 3 and Figure 4) were made with the use of a special permit issued yearly from the Greek ministry of Environment and Energy during spring and summer of 2018–2020.

In line with the provisions of the Convention of Biological Diversity and the Nagoya Protocol (www.cbd.int/abs/ (accessed on 13 March 2021)) for future utilization, a unique IPEN (International Plant Exchange Network,) number has been given for each independent collection from wild Greek populations performed (https://www.bgci.org/our-work/policy-and-advocacy/access-and-benefit-sharing/the-international-plant-exchange-network/ (accessed on 13 March 2021). Unique IPEN accession numbers were also assigned to each material received after repatriation from foreign BGs, and each purchased material from specialized nurseries.

**Figure 5:**
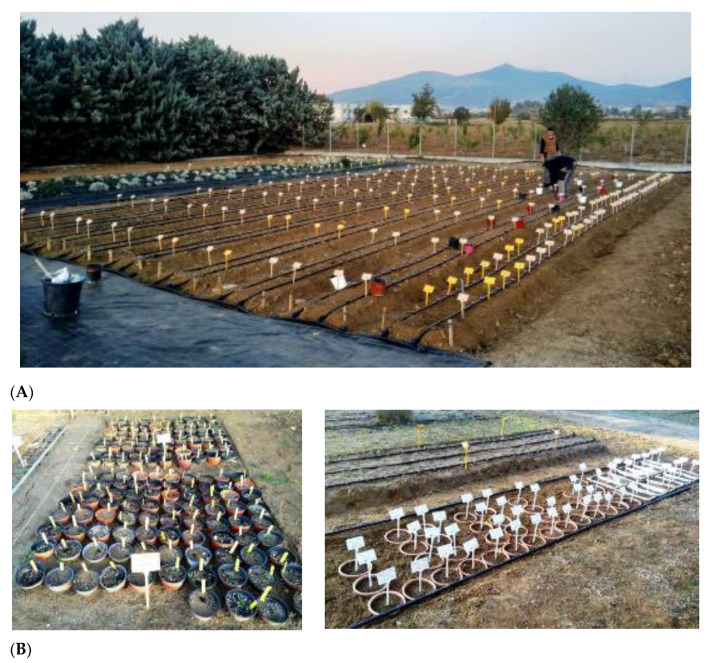
View of the 38 IPEN accessions of wild-growing Greek tulips belonging to 13 species (see Table 1) planted in the field (**A**) and in submerged pots (**B**) during autumn 2020 at the premises of the Institute of Plant Breeding and Genetic Resources in Thermi, Thessaloniki, Northern Greece for ex situ conservation and first experimentation in the frame of the research project TULIPS.GR.

All tulip bulbs with IPEN numbering have been planted outdoors during late October-mid-November in submerged pots in the ground or at the experimental field of the BBGK in Thermi, Thessaloniki, Northern Greece (Figure 5).

## 4. Conclusions

The ornamental industry is always in quest of “new” choices even for clonally propagated crops like tulips. Given the high demand for Greek tulips satisfied by 24 nurseries in four countries and the relatively high prices of the traded materials which are clonally produced, this ongoing research demonstrated that there are opportunities and potential for the introduction of new tulip species (botanical tulips) into the market [12]. At least four Greek tulip species are not traded at all over the internet, thus gaps are available in the international market. As overviewed herein with regard to Greek tulips, and apart from wild-growing populations in Greece, there are 41 botanic gardens around the world as well as 25 specialized nurseries in three countries which are detected as possible sources of propagation material for documentation, evaluation and experimentation in the frame of the project TULIPS.GR. While bulbs of almost a dozen of Greek tulips seem rather easy to obtain, seeds are comparatively harder to find and restricted to only a few species.

From a conservation viewpoint, it is evident that most of the Greek tulips are not effectively conserved in ex situ facilities (eight out of 15 species), thus suggesting the need for intense conservation efforts regarding the threatened and range-restricted species. The project TULIPS.GR aims to bridge this gap. Our study showed that the vendors of the electronic tulip trade did not provide exact information on the origin of the plants that they were selling, and therefore the possible pressure exerted on the wild-growing populations of the traded range-restricted or threatened species is currently not known [2,3,7,11].

From the legislative viewpoint, it is known that the local endemic phytogenetic resources (such as the Greek endemic *Tulipa* spp.) are subjected to sovereign rights controlling Access and Benefit Sharing policies according to the Nagoya Protocol and the EU Directive 511/2014. Furthermore, at national scale the collection and trade of local endemic plants is forbidden according to Greek law for the protection of biodiversity [3937/11 (Governmental Gazette 60/A/31-3-2011)], while the Greek Presidential Decree 67/1981 offers national protection status for all Greek tulips (endemic and non-endemic ones). Consequently, it is evident that any utilization of the Greek tulip species in the future should comply with the provisions of national and international legislation.

In this framework, the research project TULIPS.GR aims to establish for the first time in Greece a national collection for all Greek tulips that will document and explore their diversity (Figure 5), enabling all the necessary conditions for the creation of a sustainable tulip value chain. The integrated approach that is followed includes well-documented and authorized collections of wild material, mycorrhizal diversity of wild tulips, and sustainable ex situ conservation schemes, production of well-documented and DNA barcoded propagation material through species-specific propagation and cultivation protocols, e.g., [22,23], foliar diagnostics and soil/nutrient analyses, field studies in different areas, utilization of innovative precision fertilizers, and postharvest investigation of fresh cut flowers, involving networking and synergies with relevant producers and associations in Greece and beyond.

## Figures and Tables

**Table 1:** Overview of the electronic trade and ex situ conservation of the 15 wild-growing Greek tulips (chorology, endemism, and protection status according to the official Vascular flora of Greece (http://portal.cybertaxonomy.org/flora-greece/ (accessed on 13 March 2021)) as well as species’ extinction risk assessments [25,26,27] and currently acquired accessions for their sustainable exploitation in the frame of the project TULIPS.GR.

Tulips (*Tulipa* spp.)	Endemism	Extinction Risk/Protection Status	Nurseries/Countries	BGs/Countries	Accs/Wild
*T. agenensis*	Irano-Turanian	No/GPD	2/1	9/6	1/1
*T. australis*	Mediterranean-SW Asiatic	No/GPD	5/1	4/4	3/2
*T. bakeri*	Greek (Cr)	CR [27]/GPD	13/2	3/1	3/2
*T. bithynica*	Balkan-Anatolia	No/GPD	No	1/1	1/1
*T. clusiana*	Irano-Turanian	No/GPD	8/2	22/9	2/1
*T. cretica*	Greek (Cr)	EN [27], *LC/GPD	3/1	9/5	4/3
*T. doerfleri*	Greek (Cr)	CR [27], VU [26]/GPD	1/1	2/2	2/2
*T. goulimyi*	Greek (Cr, Pe)	VU [25,27]/GPD	No	2/2	4/3
*T. hageri*	Greek (StE, Pe)	EN [27], *DD/GPD	4/2	9/6	No
*T. orphanidea*	Greek (StE, Pe)	EN [27]/GPD	8/2	15/8	4/3
*T. radii*	East Mediterranean	No/GPD	1/1	2/2	3/3
*T. rhodopea*	Balkan	No/GPD	No	2/2	2/2
*T. saxatilis*	South Aegean subendemic	No/GPD	7/2	26/12	5/4
*T. scardica*	Balkan	No/GPD	No	2/2	No
*T. undulatifolia*	Balkan-Anatolia	VU [25]/GPD	2/1	8/7	4/4

Phytogeographical regions of Greece—StE: Sterea Ellada; Pe: Peloponnese; Cr: Crete; CR: Critically Endangered; EN: Endangered; VU: vulnerable; DD: data deficient; LC: least concern (according to criteria of the International Union for the Conservation of Nature) [25,26,27]; * Global IUCN Red List (www.iucnredlist.org (accessed on 13 March 2021)); No: absence of information; GPD: Included in the Greek Presidential Decree 67/1981; BG: Botanic gardens; Accs: Total accessions documented; Wild: independent collections from a wild-growing population in a given locality.

## Data Availability

The datasets generated and/or analyzed during the current study are available from the corresponding author on request.
